# Cannabidiol Protects Dopaminergic Neuronal Cells from Cadmium

**DOI:** 10.3390/ijerph16224420

**Published:** 2019-11-12

**Authors:** Jacopo Junio Valerio Branca, Gabriele Morucci, Matteo Becatti, Donatello Carrino, Carla Ghelardini, Massimo Gulisano, Lorenzo Di Cesare Mannelli, Alessandra Pacini

**Affiliations:** 1 Department of Experimental and Clinical Medicine, Histology and Anatomy Section, University of Firenze, 50134 Firenze, Italy; donatello.carrino@unifi.it (D.C.); massimo.gulisano@unifi.it (M.G.); alessandra.pacini@unifi.it (A.P.); 2 Department of Experimental and Clinical Biomedical Sciences “Mario Serio”, University of Firenze, 50134 Firenze, Italy; matteo.becatti@unifi.it; 3 Department of Neuroscience, Psychology, Drug Research and Child Health (NEUROFARBA), Pharmacology and Toxicology Section, University of Firenze, 50139 Firenze, Italy; carla.ghelardini@unifi.it (C.G.); lorenzo.mannelli@unifi.it (L.D.C.M.)

**Keywords:** cadmium, cannabidiol, ER stress, ROS, SH-SY5Y, neurotoxicity

## Abstract

The protective effect of cannabidiol (CBD), the non-psychoactive component of *Cannabis sativa*, against neuronal toxicity induced by cadmium chloride (CdCl_2_ 10 μM) was investigated in a retinoic acid (RA)-differentiated SH-SY5Y neuroblastoma cell line. CBD (1 μM) was applied 24 h before and removed during cadmium (Cd) treatment. In differentiated neuronal cells, CBD significantly reduced the Cd-dependent decrease of cell viability, and the rapid reactive oxygen species (ROS) increase. CBD significantly prevented the endoplasmic reticulum (ER) stress (GRP78 increase) and the subcellular distribution of the cytochrome C, as well as the overexpression of the pro-apoptotic protein BAX. Immunocytochemical analysis as well as quantitative protein evaluation by western blotting revealed that CBD partially counteracted the depletion of the growth associated protein 43 (GAP43) and of the neuronal specific class III β-tubulin (β3 tubulin) induced by Cd treatment. These data showed that Cd-induced neuronal injury was ameliorated by CBD treatment and it was concluded that CBD may represent a potential option to protect neuronal cells from the detrimental effects of Cd toxicity.

## 1. Introduction

Cadmium (Cd) is a transition heavy metal, chemically similar to zinc and mercury, the two other metals in group 12, whose preferential oxidation state is +2. As an important component of industrial processes such as metal plating, production of nickel-cadmium batteries, pigments, plastics, and other synthetics, Cd has been seen as an occupational hazard [1]. On the other hand, tobacco smoking, air pollution, and consumption of Cd-contaminated drinking water are the major sources of non-occupational Cd exposure [2,3]. If the primary route of exposure in industrial settings is inhalation of Cd-containing fumes, food and water are generally the largest sources of Cd exposure in a nonsmoker population [4]. Epidemiological and experimental studies have linked the occupational Cd exposure with lung cancer and other cancers such as the prostate, renal, liver, hematopoietic system, urinary bladder, pancreatic, testis, and stomach cancers [5,6,7].

Exposure to Cd also severely affects the function of the nervous system [8,9,10,11], with symptoms including headache and vertigo, olfactory and motor dysfunction, peripheral neuropathy, decreased equilibrium and ability to concentrate, and learning disabilities [12,13,14,15]. Although studies on the central nervous system (CNS) Cd distribution demonstrated that this metal could not easily get into the brain due to the presence of the blood brain barrier (BBB) [16,17], a Cd-induced BBB dysfunction and permeability increase has been demonstrated in in vivo models [18]. Also, authors reported the presence of BBB impairment in several neurodegenerative diseases, such as Alzheimer’s disease (AD) and Parkinson’s disease (PD) [19,20]. Cd was shown to adversely influence the functions of cholinergic and catecholaminergic systems [21], as well as the balance between excitation–inhibition in synaptic neurotransmission [22]. Also, it has been proposed that chronic exposure to Cd can be associated with an increased risk of developing PD [23]. Parkinson’s disease is a neurodegenerative condition characterized by loss of dopaminergic neurons in the *substantia nigra pars compacta* with resulting neurochemical imbalance throughout the basal ganglia [24]. Interestingly, this heavy metal shows similar mechanisms of toxicity with other pollutants: they accumulate in the *substantia nigra* and generate oxidative stress by increasing the production of reactive oxygen species (ROS) and/or deregulating the antioxidant enzymes. This, in turn, produces the activation of the glia inducing neuroinflammation, which increases the generation of further oxidative stress, leading to a self-perpetuating cycle [25,26].

Although the mechanisms of Cd toxicity are poorly understood, the neurotoxicity of Cd is attributable to the generation of ROS. Oxidative stress is generally defined as an imbalance that favors the production of ROS and reactive nitrogen species (RNS) [27,28,29]. The major consequence induced by Cd through oxidative stress is a ROS-mediated attack of double bonds in membrane lipids that results in increased lipid peroxidation (LPO) as well as interference with the endogenous antioxidant defenses in several organs and systems [27,30,31,32,33]. Indeed, Cd is known to induce a mitochondrial membrane potential decrease and the consequent release of cytochrome *C*, eventually leading to the activation of caspase-3 [34]. Furthermore, it has been demonstrated that Cd induces ER stress [21,35].

Previous in vitro and in vivo studies showed that Cd neurotoxicity was significantly attenuated by antioxidants, anti-inflammatory, and metal-chelating agents [21,36,37,38,39]. *Cannabis sativa* has been used for medicinal/recreational purposes for many years [40]. The two major components are Δ^9^-tetrahydrocannabinol (Δ^9^-THC), the main psychoactive ingredient, and cannabidiol (CBD), the major non-psychoactive component [41,42,43].

The adverse effects of cannabis are attributed to Δ^9^-THC [44], whereas CBD exhibits a variety of therapeutic properties: anti-inflammatory, antidepressant, anxiolytic, immunomodulatory, antioxidant, and neuroprotective effects [45,46,47,48,49,50,51,52]. 

CBD has been shown to reverse the increased excitotoxicity, inflammation, and oxidative stress in ischemic brain damage and to protect PC12 and SH-SY5Y cells from tert-butyl-hydroperoxide-induced oxidative stress [53]. Also, recent studies demonstrated that CBD was able to reverse the reductions in synaptophysin levels and increases in caspase-3 levels induced by iron [54].

For this purpose, SH-SY5Y cell line presents many advantages including that can be differentiated using retinoic acid (RA). Recently, Korecka and colleagues [55] have characterized the molecular phenotype of RA-differentiated SH-SY5Y cells and concluded that these cells exhibit a dopaminergic phenotype. The use of differentiated SH-SY5Y cells is well established as a cell culture model of PD [56]. 

In the present study, we provide evidence that CBD offers protection to neuronal cells against Cd-induced oxidative stress by decreasing ROS production. Finally, we demonstrate the protective effects of CBD against Cd-induced ER stress, pro-apoptotic BAX upregulation, cytochrome C release, and the modifications in the expression levels and in the cellular distribution of the growth associated protein 43 (GAP43) and of the neuronal specific class III β-tubulin (β3 tubulin), two proteins involved in the neuronal sprouting.

## 2. Materials and Methods 

### 2.1. Cell Line 

Human neuroblastoma SH-SY5Y cell line, was purchased by Istituto Zooprofilattico Sperimentale della Lombardia e dell’Emilia-Romagna (Brescia, Italy). Cells were routinely cultured in DMEM High Glucose/Ham’s F12 Mixture Medium (1:1 ratio), adding 10% fetal bovine serum (FBS), 2 mM L-Glutamine (EuroClone S.p.a., Milano, Italy) and maintained at 37 °C, 5% CO_2_ in humidified atmosphere. The growth medium was changed every 2–3 days.

In all the experiments pointed in this paper, SH-SY5Y cells were differentiated with 10 μM all-trans retinoic acid (RA) (Sigma Aldrich, Milano, Italy) for 48 h in their appropriate medium (DMEM High Glucose/Ham’s F12 Mixture Medium (1:1), 2 mM L-Glutamine) supplemented with 1% FBS, as previously reported [21]. The 48 h differentiation was established because a prolonged SH-SY5Y RA treatment induces cells to release cytochrome C from mitochondria to cytoplasm [57,58]. Briefly, the cells were seeded in each support for 24 h in their complete growth medium. The next day, cells were starved in 1% FBS medium for 48 h and differentiated by adding RA 10 μM. After two days of differentiation, the cells were starved in 0% FBS medium for 24 h and then stimulated at different times in starved medium (0% FBS) as reported below. 

### 2.2. Treatment

In order to reproduce in vitro conditions that could mimic a chronic human Cd intoxication, we decide to use a concentration of 10 μM of cadmium chloride (CdCl_2_) (Sigma Aldrich, Milano, Italy) and a time of exposure of 24 h, as previously reported [21,59].

With the aim to figure out the neuro-protective effects of CBD, the concentration of 1 µM was chosen on the basis of previously reported data [60] and performing dose response curves (data not shown).

Furthermore, since CBD has been evaluated in literature for its antioxidant effects [46,61], we compared the CBD actions with αTocopheryl acetate (αToco) at 10 µM [62]. 

All treatments were performed in starvation medium because Cd effects could be impaired by the presence of essential elements (such as zinc and calcium) in FBS. The timeline with the entire experimental procedures was the following: 

CdCl_2_ 10 µM was placed in a starvation medium for 24 h. For pre-treatment experiments, CBD 1 µM or αToco 10 µM were added into the starvation medium for 24 h. The following day, the medium was replaced with CdCl_2_ 10 µM in the starvation medium for another 24 h.

### 2.3. MTT Assay

Cell viability was evaluated by the reduction of 3-(4,5-di-methyl-thiozol-2-yl)-2,5-diphenyltetrazolium bromide (MTT) as an index of mitochondrial functional activity. Briefly, SH-SY5Y cells were seeded into 96 well plates at a density of 20,000 cells/well in complete growth medium for one day and RA differentiated. After differentiation, cells were treated with or without CdCl_2_ after the pre-treatment with CBD or αToco. After removing the medium with different stimuli, 1 mg/ml MTT was added into each well and incubated for at least 20 min at 37 °C. Following this, the chromogenic solution was removed and replaced with 50 µL of dimethyl sulfoxide (DMSO) to dissolve the formazan crystals, and the absorbance was measured at 595 nm wavelength by a Multiscan FC photometer (ThermoFisher Scientific, Milano, Italy). Three independent experiments were performed, and each experiment was performed in quintuplicate.

### 2.4. ROS Evaluation

To assay the intracellular ROS production, 7 × 10^4^ SH-SY5Y cells were seeded on square (22 × 22 mm) glass cover slip slides and lodged in 6 multi-well plates. After RA differentiation and CdCl_2_, CBD, αToco appropriate stimulation, cells were loaded with 10 μM 2,7-dichlorodihydro-fluorescein diacetate for 10 min (CM-H_2_DCFDA, ThermoFisher Scientific, Milano, Italy), as previously described [63]. Cell fluorescence was captured by the motorized Leica DM6000B microscope equipped with a DFC350FX (Leica, Mannheim, Germany). The microscope was set at optimal acquisition conditions, and settings were kept constant for each analysis. Five microscopic fields for each experimental point were analyzed. 

Fluorescence intensity was processed by ImageJ analysis software (ImageJ, National Institute of Health, Bethesda, MD, USA, https://imagej.nih.gov/ij/, version 1.52 q) and the results were expressed as a percentage of control.

Each experiment was performed in triplicate, and three different experiments were set up. Furthermore, a positive control was performed treating SH-SY5Y with H_2_O_2_ (Sigma Aldrich, Milano, Italy) 200 µM for 24 h (supplementary Figure S1).

### 2.5. Western Blotting Analysis

SH-SY5Y cells were plated in Petri dishes in complete growth medium at the density of 10^7^ cells/well and treated as described above. After each treatment, the medium was removed and two washes with PBS (phosphate buffered saline) were performed. The cells were scraped from the surface of the dishes and the cell suspensions were centrifuged at 1000 rpm for 10 min at room temperature (RT). After removing the supernatant, the pellets were treated with lysis buffer (TRIS 50 mM, pH 7; NaCl 150 mM; 1% TRITON X-100; EDTA 1.5 mM; 0.25% SDS) containing a protease inhibitors cocktail (Sigma Aldrich, Milano, Italy) for 30 min at 4 °C. The homogenates were centrifuged at 4 °C for 10 min at 12,000 rpm and the obtained supernatants were used to evaluate the protein concentration by Bradford’s method. Equal amounts of proteins (30 μg) were analyzed on a 12–14% polyacrylamide gel, subjected to protein molecular weight, and then transferred onto nitrocellulose membrane (Porablot NPC, MACHEREY-NAGEL, Milano, Italy). After 1 h blocking with 3% bovine serum albumin (BSA) in Tris-buffered saline containing 0.1% Tween 20 (T-TBS) at RT, the blot was incubated overnight at 4 °C with the following primary antibodies: 1:500 mouse monoclonal anti-GAP43 (B-5), 1:10,000 mouse monoclonal anti-β-actin (C4), 1:300 rabbit polyclonal anti-BAX (P-19), 1:500 rabbit polyclonal anti-cytochrome C (H-104) (Santa Cruz Biotechnology, Santa Cruz, CA, USA), 1:500 rabbit polyclonal anti-GRP78 antibody (ThermoFischer Scientific, Milano, Italy), 1:10,000 rabbit polyclonal anti-GAPDH (Cell Signaling, Boston, MA, USA), and then with 1:5000 goat anti-mouse (for GAP43 and β-actin) and goat anti-rabbit (for BAX, cytochrome C, GRP78 and GAPDH) HRP secondary antibodies (Santa Cruz Biotechnology, Santa Cruz, CA, USA) for 1 h at RT. The protein amounts were detected with the Amersham ECL Plus Western Blotting Detection Reagent (GE Healthcare, Milano, Italy). Protein expression levels were then quantified by ImageJ analysis software and expressed as a percentage of control.

Each experiment was performed in triplicate, and three different experiments were performed.

### 2.6. Immunofluorescent Staining

SH-SY5Y cells were seeded on square (22 × 22 mm) glass cover slip slides lodged in 6 multi-well plates at the density of 7 × 10^4^ cells/well. As described above, RA differentiated SH-SY5Y were treated with CdCl_2_ with or without CBD or αToco. At the end of each treatment, the starvation medium containing stimuli was removed and two washes with cold PBS were performed. The cells were then fixed with 1 mL of 4% paraformaldehyde for 10 min at RT. After three more washes with cold PBS (5 min for each wash), the cells were permeabilized with 0.1% TRITON X-100 in PBS for 10 min at RT. Cells were then washed three times with PBS and incubated for at least 15 min in a blocking solution (1% BSA in PBS) at RT. Then, each cover slip was incubated overnight at 4 °C with the following primary antibodies at 1:200 dilution: mouse monoclonal anti-β3 tubulin (TU-20), mouse monoclonal anti-GAP43 (B-5), rabbit polyclonal anti-cytochrome C (H-104) (Santa Cruz Biotechnology, Santa Cruz, CA, USA). The day after, cells were washed three times with PBS and each cover slip was incubated with 1:200 dilution Alexa Fluor 568 goat anti-mouse (for β3 tubulin and GAP43) or 488 goat anti-rabbit (for cytochrome C) immunoglobulin G (IgG) secondary antibodies (ThermoFisher Scientific, Milano, Italy) for 1 h at RT. After secondary antibody incubation, two washes were performed with PBS and DAPI (4′,6-dia-midin-2-fenilindolo; 1:2000 dilution; ThermoFisher Scientific, Milano, Italy) was added for 5 min at RT to each cover slip. Eventually, after two more washes in cold PBS and one more in distilled water, cover slip glasses were mounted by Fluoromount anti-fade solution (ThermoFischer Scientific, Milano, Italy) on cover slides. Digitalized images were collected at 200× or 400× total magnification by a motorized Leica DM6000B microscope equipped with a DFC350FX. Five microscopic fields for each experimental point were analyzed.

All data were reported after normalization for the total number of cells per field. Each experiment was performed in triplicate, and three different experiments were set up.

### 2.7. Statistical Analysis

Statistical analysis was performed by Two-way Analysis of Variance (ANOVA) followed by the Tukey test. All assessments were made by researchers blinded to treatments. Data were analyzed using “Origin 9” software (OriginLab, Northampton, MA, USA). Differences were considered significant at *p* < 0.05.

## 3. Results

### 3.1. Cell Viability

**Figure 1:**
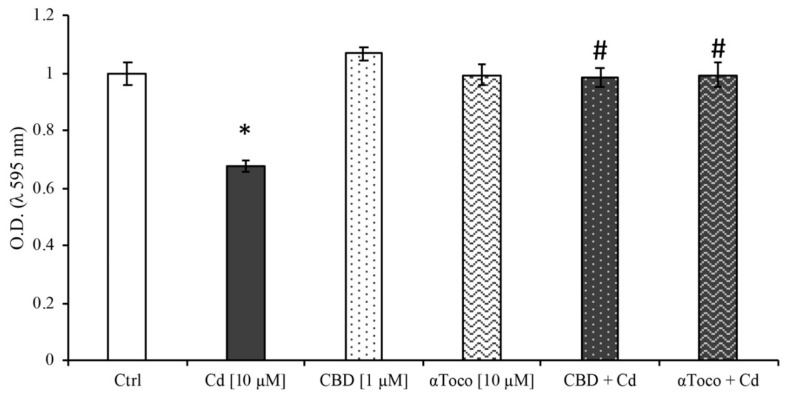
*Cell viability on retinoic acid (RA) differentiated SH-SY5Y*. An 3-(4,5-di-methyl-thiozol-2-yl)-2,5-diphenyltetrazolium bromide (MTT) assay was used to evaluate CdCl_2_ 10 µM effects after cannabidiol (CBD) 1 µM or αToco 10 µM pre-treatment. As expected, a Cd-dependent significant reduction of cell viability was observed (dark column). Dissimilarly, both CBD (dotted column) and αToco (wavy column) clearly prevented Cd-induced neurotoxicity. Results are expressed as mean value ± S.E.M. Control, untreated cells, were taken as 100%. Each experimental point was performed in quintuplicate, from three independent set of experiments. * *p* < 0.05 vs. Ctrl; # *p* < 0.05 vs. Cd.

To evaluate RA differentiated SH-SY5Y cell viability after treatments with CdCl_2_ 10 µM with or without CBD 1 µM and αToco 10 µM, an MTT assay was performed. As shown in Figure 1, cell viability was significantly (* *p* < 0.05) decreased after 24 h treatment with CdCl_2_. In contrast, both CBD and αToco were able to prevent the decrease of cell viability when used as pre-treatment for 24 h before adding CdCl_2_.

### 3.2. SH-SY5Y ROS Production

**Figure 2:**
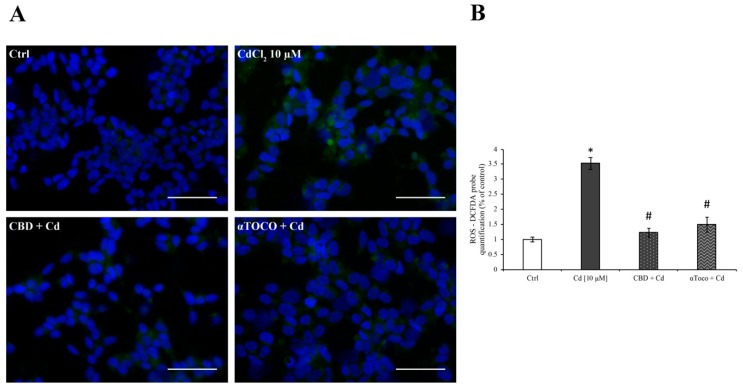
*Immunofluorescence staining and ROS production quantification.* ROS expression was quantified by the 2′,7′-dichlorodihydrofluorescein diacetate (CM-H_2_DCFDA) probe. (**A**) Representative fluorescent images of intracellular ROS levels after CdCl_2_ 10 µM treatment, with or without CBD or αToco pre-treatment. (**B**) Fluorescence semi-quantitative analysis highlighting the role of Cd (dark column) in increasing ROS production and the role of CBD (dotted column) or αToco (wavy column) in preventing ROS formation. The intracellular ROS-derived fluorescence is expressed as the percentage of fluorescence compared to control, untreated cells, taken as 100%. Results are expressed as mean ± S.E.M. Each experiment point was performed in triplicate, from three different set of experiments. Five different microscopic fields for each experimental point were analyzed. Total magnification: 400×. Scale bar: 50 μm. * *p* < 0.05 vs. Ctrl; # *p* < 0.05 vs. Cd.

In order to evaluate the effects of CBD and αToco on Cd-induced ROS generation leading to oxidative stress, RA differentiated SH-SY5Y cells were treated with 10 μM CdCl_2_ for 24 h after the pre-treatment with or without CBD 1 µM and αToco 10 µM. The fluorescent images (Figure 2A), clearly showed that CdCl_2_ increased the amounts of ROS, confirming the oxidative role of Cd leading to neuronal toxicity, as previously reported [64]. On the other hand, both CBD and αToco seemed to be protective against Cd-induced oxidative stress. The semi-quantitative analysis of ROS levels (Figure 2B), better highlighted the Cd-induced ROS increment, as well as the effectiveness of CBD or αToco in RA differentiated SH-SY5Y.

### 3.3. Cd-Induced ER Stress and Apoptotic Cascade Signaling

**Figure 3:**
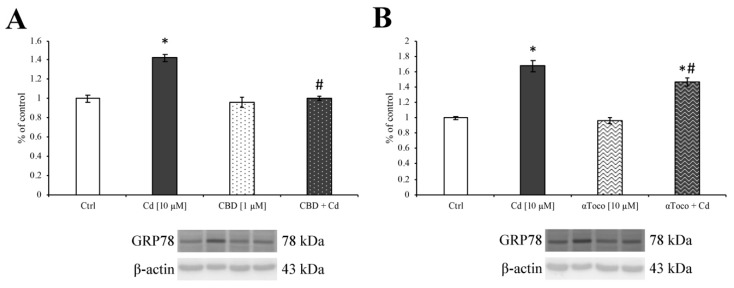
*Western blotting analysis on ER stress.* Western blotting analysis showed that CdCl_2_ 10 µM significantly increased SH-SY5Y ER stress, by the up-regulation of GRP78 (dark columns). On the other hand, both CBD 1 µM (**A**—dark dotted column) and αToco 10 µM (**B**—dark wavy column) pre-treatment, significantly prevented the ER stress retaining GRP78 expression levels to control amounts. Results are expressed as mean ± S.E.M. Control, untreated cells, were taken as 100%. The housekeeping β-actin protein was used as an internal control for protein normalization. Each experiment point was performed in triplicate, from three different set of experiments. * *p* < 0.05 vs. Ctrl; # *p* < 0.05 vs. Cd.

To better figure out the ER stress induced by the presence of Cd, we evaluated the expression of GRP78, a HSP70 molecular chaperone well known to be evoked during ER stress [65]. In Figure 3A,B, the histogram clearly shows how CdCl_2_ (dark columns) treatment is able to over-express GRP78. This deleterious effect was prevented by the pre-treatment with CBD (Figure 3A, dark dotted column) and αToco (Figure 3B, wavy dark column) as well.

**Figure 4:**
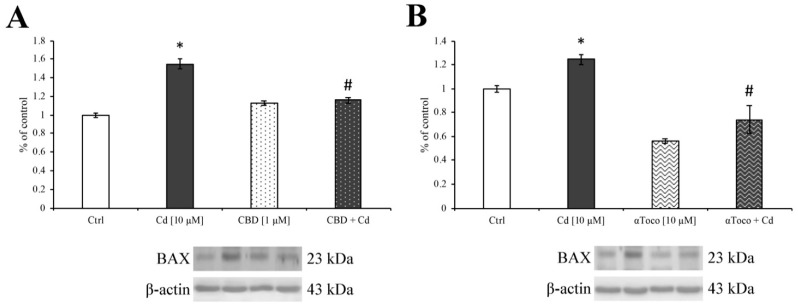
*Western blotting analysis on pro-apoptotic protein.* Consistent with Cd-induced SH-SY5Y ER stress, the histograms clearly showed a significant increase in BAX protein expression after 24 h CdCl_2_ treatment (dark columns). The pre-treatment with CBD 1 µM (**A**—dark dotted column) and αToco 10 µM (**B**—dark wavy column), were able to significantly retain low level of the pro-apoptotic BAX protein as reported by western blotting analysis. Results are expressed as mean ± S.E.M. Control, untreated cells, were taken as 100%. The housekeeping β-actin protein was used as an internal control for protein normalization. Each experiment point was performed in triplicate, from three different set of experiments. * *p* < 0.05 vs. Ctrl; # *p* < 0.05 vs. Cd.

Moreover, since Cd is well known to activate the apoptotic pathway [34], the expression levels of the pro-apoptotic protein BAX (Figure 4A,B), were investigated during CBD and αToco pre-treatment in RA differentiated SH-SY5Y. Our results undoubtedly showed an increment in BAX expression level after 24 h CdCl_2_ (dark columns) treatment, while both CBD and αToco pre-treatment showed a protective effect, preserving low BAX levels superimposable to control amounts.

**Figure 5:**
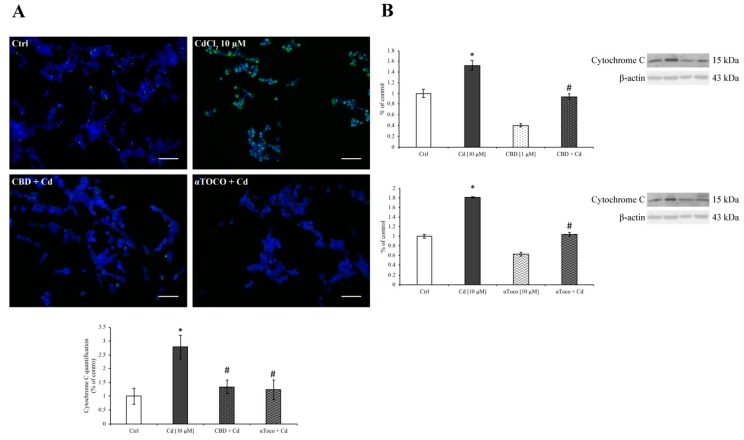
*Cytochrome C expression on SH-SY5Y*. (**A**) Immunofluorescent images of cytochrome C are shown. During CdCl_2_ treatment, a conspicuous increment of cytoplasmic cytochrome C was observed. In contrast, the presence of CBD or αToco prevented such an increase in the cell soma. These results were also highlighted by the histogram. (**B**) Cytochrome C expression levels evaluated by western blotting analysis. As reported, CdCl_2_ (dark columns) treatment significantly increased the protein expression after 24 h of treatment. On the other hand, CBD (dotted dark column) or αToco (wavy dark column) retained cytochrome C up-regulation. Results are expressed as mean ± S.E.M. Control, untreated cells, were taken as 100%. The housekeeping β-actin protein was used as an internal control for protein normalization. Each experiment point was performed in triplicate, from three different sets of experiments. Total magnification: 200×. Scale bar: 50 μm. * *p* < 0.05 vs. Ctrl; # *p* < 0.05 vs. Cd.

These results collimate with the mitochondrial spillage of cytochrome C in the cytoplasm. Indeed, as displayed in Figure 5A, the immunofluorescent staining of cytochrome C revealed an increase during 24 h CdCl_2_ treatment. Nevertheless, when RA differentiated SH-SY5Y were pre-treated with CBD or αToco, the cytochrome C remained poorly labeled close to the nuclei as seen in control, untreated cells. This last result was highlighted by western blotting analysis (Figure 5B). The densitometric analysis of cytoplasm protein expression levels revealed a high amount of cytochrome C during CdCl_2_ (dark columns) treatment, which was retained at a lower level when RA differentiated SH-SY5Y were in the presence of CBD (dotted dark column) or αToco (wavy dark column).

### 3.4. Neuronal Sprouting

**Figure 6:**
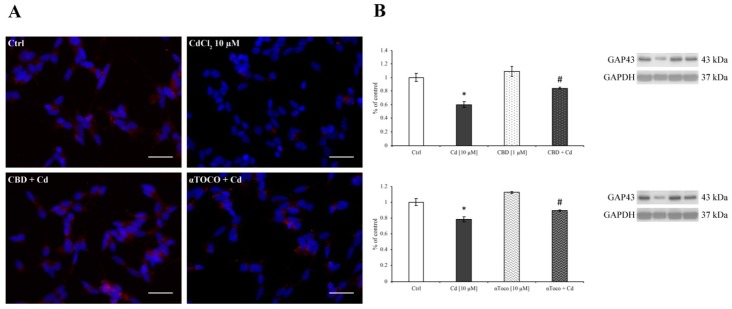
*GAP43 expression on SH-SY5Y*. (**A**) Immunofluorescent images of GAP43. As shown, a significant decrease was observed after CdCl_2_ treatment. Differently, when SH-SY5Y were pre-treated with CDB or αToco, the GAP43 levels were superimposable on control, untreated cells. (**B**) GAP43 expression levels evaluated by western blotting analysis. As expected, CdCl_2_ (dark columns) treatment significantly decreased the protein expression after 24 h treatment. On the other hand, CBD (dotted dark column) or αToco (wavy dark column) were able to preserve the Cd-dependent GAP43 down-regulation expression levels. Results are expressed as mean ± S.E.M. Control, untreated cells, were taken as 100%. The housekeeping GAPDH protein was used as an internal control for protein normalization. Each experiment point was performed in triplicate, from three different set of experiments. Total magnification: 400×. Scale bar: 25 μm. * *p* < 0.05 vs. Ctrl; # *p* < 0.05 vs. Cd.

These detrimental effects induced by Cd may affect neuronal sprouting. For this reason, we evaluated the expression of GAP43 in RA differentiated SH-SY5Y cells during CdCl_2_ treatment in the presence or absence of CBD and αToco. As shown in Figure 6A, Cd-treated cells express low levels of perinuclear GAP43, compared to control, untreated cells. On the other hand, when our cellular model was pre-treated with CBD or αToco, the immunofluorescent staining revealed a higher presence of GAP43 that was maintained at control amounts. Furthermore, western blotting analysis (Figure 6B), completely correlate with immunofluorescent staining performed. Indeed, since CdCl_2_ (dark columns) treatment decrease GAP43 expression, CBD (dotted dark column) and αToco (wavy dark column) pre-treatment were able to prevent the GAP43 decrease, retaining levels that are superimposable to control amounts. Taken together, these results encouraged us to better investigate the protective role of CBD in neuronal branching, looking at β3 tubulin.

**Figure 7:**
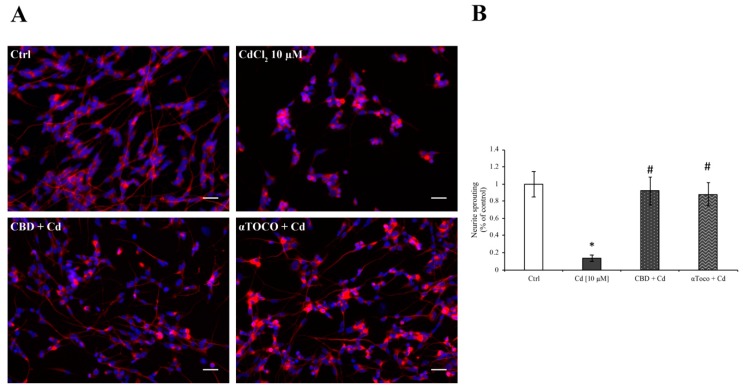
*β3 tubulin expression on SH-SY5Y*. (**A**) the immunofluorescence staining revealed a very strong decrease of cytoplasmic elongations in CdCl_2_-treated cells. This deleterious event was prevented by the presence of CBD 1 µM or αΤoco 10 µM. (**B**) highlights the number of neurite elongations, normalized on nuclei count. The results are expressed as mean ± S.E.M. and control, untreated cells are reported as 100%. The average number of cytoplasmic elongations were the following: Ctrl (197.4 ± 29.3), CdCl_2_ 10 µM (27.2 ± 6.8), CBD + Cd (181.4 ± 31.7), αTOCO + Cd (173.9 ± 26.4). Each experiment point was performed in triplicate, from three different set of experiments. Total magnification: 200×. Scale bar: 50 µm.

The immunofluorescent staining of β3 tubulin (Figure 7A) corroborate and support the GAP43 expression. Indeed, the cytoplasmic elongation clearly decreased when cells were treated with CdCl_2_ 10 µM for 24 h, and β3 tubulin appeared abundant in the cell soma, alongside the nuclei. Interestingly, when RA differentiated SH-SY5Y were pre-treated with CBD or αToco, the neuronal sprouting was maintained, hypothesizing the protective role of CBD against the Cd neuro-toxic effects and neurite loss. To better underline the positive role of CBD in counteracting Cd neurite loss, the occurrence of neuronal sprouting was quantified by Fiji software (ImageJ, Bethesda, MD, USA), and then normalized on nuclei counting (Figure 7B). 

## 4. Discussion

In recent years, there has been an increasing ecological and global public health concern associated with environmental contamination by heavy metals. As a result of an exponential increase of their use in several industrial, agricultural, domestic, and technological applications, human exposure to these metals has risen dramatically [66]. Although heavy metals are naturally occurring elements, most environmental contamination and human exposure result from anthropogenic activities such as mining and smelting operations, industrial production and use, and domestic and agricultural use of metals and metal-containing compounds [67,68,69,70].

Cadmium is a heavy metal of considerable environmental and occupational concern [71]. Human exposure to Cd is possible through a number of several sources including employment in primary metal industries, emissions from industrial activities, including mining, smelting, and manufacturing of batteries, pigments, stabilizers, and alloys [72].

Agricultural and industrial activities have led to the entry of Cd into the soil and subsequently into ground and drinking water. Due to the highly soluble nature of Cd compounds compared to other metals, it is readily taken up by plants, resulting in storage in crops for food and feed production. This high soil-to-plant transfer rate makes the diet, in general, the primary source of Cd exposure in non-occupationally exposed populations [4].

Occupational exposure to Cd takes place in industrial factories, where it is frequently used [73], such as from zinc (Zn) smelters, battery manufacturing and metal recovering factories, Cd refining companies, paint, and pigment production units, as well as via other anthropogenic factors like waste incineration and fossil fuel combustion [30].

Once absorbed, Cd is reported to accumulate in several tissues, which might cause harmful effects including renal dysfunction, pulmonary edema, several different organ cancers, cardiovascular disease, airway inflammation, diabetes, and neurological diseases [74]. Cd has been regarded as a possible etiological factor for human neurodegenerative diseases, such as PD and AD [23]. Indeed, over the last several years many studies have extensively demonstrated that exposure to different environmental factors could be a significant risk factor for the development of PD [25,75]. Even if the etiology of this disease is still unclear, the role of the environment as a putative risk factor has gained importance. Moreover, neurotoxic metals, including Cd, have been involved in PD [23].

Normal blood plasma Cd concentrations in adults without excessive exposure are generally 8–30 nM [76,77], other authors consider as normal the range 1.8–50 nM [78]; recently the World Health Organization (WHO) [71] reported that an acceptable blood level of Cd is in the range of 2–10 nM. Concentrations above 0.045 µM are warrant careful investigation [79]. About the definition of a toxic blood levels there are some discrepancies; in 1980, Kaye reported that the approximate lethal blood level is 4.5 µM [77]. A recent paper regarding the blood Cd levels of Nigerian subjects exposed to this metal were showed that the mean blood value was 4 µM without declared toxic effects but only “increased health risk” [80]. Acute poisoning with marked lethargy and fever was reported with blood Cd concentration 0.25 µM [78].

In vitro data are quite different, where 0.5 µM is considered an ultra-low Cd concentration able to attenuate angiogenesis in both the wound healing assay and the chick chorioallantoic membrane (CAM) assay. In addition, the same concentration of Cd reduces bradykinin (BK), a powerful angiogenic agent, and mediate both tube formation in 3D matrigel matrix and ex vivo angiogenesis in CAM models, suggesting a protective role of Cd against tumor angiogenesis [81].

On the basis of this evidence, concentrations ranging from 1 to 10 µM (one of those used in the present work) can be considered relevant for mimicking Cd-mediated damage of tissues or body compartments [59]. Moreover, based on previously reported data [82,83,84] we adopted the best experimental conditions that could better reflect an in vivo situation, thereby mimicking chronic exposure conditions. For this reason, we chose the concentration of 10 µM that was low cytolytic but, at the same time, enabled us to study the Cd effects at the cellular level.

CBD is the second major component of the *Cannabis sativa* and, with respect to the first component Δ^9^-THC, is not associated with psychoactivity. CBD possesses numerous *per se* pharmacological effects, reducing the collateral effects of Δ^9^-THC, thus ameliorating its safety profile [85,86]. Moreover, it displays lower CB_1_ and CB_2_ receptor affinity with respect to Δ^9^-THC. CB_1_ receptors are expressed most densely in the CNS and are largely responsible for mediating the effects of cannabinoid binding in the brain [87,88,89]. CB_2_ receptors are preferentially expressed in the immune and gastrointestinal systems, even if a very low expression has been identified in some neurons within the CNS (e.g., the brainstem) [90,91]. CBD also interacts with several other recently discovered CB receptors, and it is an agonist for the 5-HT_1A_ receptor [92,93], which may explain some of the antipsychotic and anxiolytic effects of CBD [94]. Dopamine receptors have been identified as other targets of CBD, in particular it is a partial agonist of the D2 receptor (D_2_R) [95]. Also, the highly lipophilic nature of cannabinoids allows CBD to enter freely the cells [96].

The mechanisms by which CBD exerts its effect are not precisely known, but it is clear that the pharmacological actions of CBD follow from many different mechanisms [43,97].

Many studies with CBD used in vivo models [53] but the evaluation of the effects of CBD on neurobehavioral and neuropathological processes is expensive, time consuming, and is not suitable to predict human neurotoxicity. Cellular models are instrumental for in vitro or ex vivo studies to analyze the cellular pathways that govern physiological or pathological processes, or to evaluate the cell toxicity or protection induced by different compounds, including potential drugs. 

In the present study, we have investigated the protective effect of treatment with CBD on RA-differentiated SH-SY5Y neuroblastoma cells that show a dopaminergic-like phenotype. These cells not only provide a human cell culture model of PD, but also possess important characteristics that make them particularly useful for analyzing the effects of CBD against the neurotoxicity induced by Cd. Indeed, it has been already demonstrated that neuronal SH-SY5Y cells selectively express the CB_1_ receptor [98]. Moreover, RA-dependent neuronal differentiation was associated with a strong induction of CB_1_R [99] and a strong increase in dopamine receptor subtypes (D_2_R and D_3_R), and dopamine transporter (DAT) expression [100,101,102]. 

Moreover, the effects of CBD were then compared with (αToco), a known antioxidant.

Our results, consistent with the antioxidant properties attributed to this compound in different experimental models [103], demonstrate that the exposure of RA-differentiated neuroblastoma cells to Cd induced intracellular ROS production at 24 h post treatment. By comparison, CBD pre-treatment reduced the Cd-dependent ROS increment, acting as ROS scavenger.

Moreover, Cd induced the release of cytochrome C from mitochondria and the upregulation of the pro-apoptotic protein BAX expression levels. These effects, already proven to be a consequence of the ROS formation [104,105,106,107], are partially prevented by the action of CBD.

Given the notion that oxidative stress triggered ER stress [108], we measured the expression levels of GRP78, a known unfolded protein response (UPR)-related chaperone [65,109,110,111] in the presence and in the absence of CBD. We observed that after 24 h of exposure to Cd the presence of CBD significantly prevented the Cd-dependent GRP78 upregulation. This result is interesting when compared with αToco that slightly counteracts Cd-induced ER stress. Indeed, CBD was seen to exploit this effect both as an anti-oxidant and as an ER stress attenuator [112,113], on the other hand, αToco mainly acts as an anti-oxidant molecule.

Finally, as previously demonstrated [21], Cd is able to induce a downregulation of GAP43 expression and an altered distribution of β3 tubulin, two proteins involved in neurite outgrowth and neurogenesis and axon guidance and maintenance, respectively. This effect is prevented by the CBD treatment.

In summary, the findings presented here indicate that CBD exerts protective effects in RA-differentiated SH-SY5Y cells under Cd treatment, a condition of oxidative and ER stress. In conditions of oxidative stress, the protective effect of CBD was mediated by a decrease in ROS production; also, CBD combated apoptosis by decreasing cytochrome C mitochondrial extrusion and downregulating BAX, and avoids ER stress through the modulation of GRP78 protein. Finally, CBD prevents the Cd-dependent inhibition of neuronal sprouting, as determined by the evaluation of GAP43 expression levels and β3 tubulin intracellular distribution. We propose that CBD, a *Cannabis sativa* derivative that lacks psychoactive properties, is a good candidate provide protection from Cd-induced neurotoxic threats.

## Supplementary Materials

The following are available online at https://www.mdpi.com/1660-4601/16/22/4420/s1, Figure S1: Immunofluorescence staining of ROS positive control.
