# Supplementary material for: Cannabidiol Protects Dopaminergic Neuronal Cells from Cadmium
Source: Int J Environ Res Public Health. 2019 Nov 12;16(22):4420. doi: 10.3390/ijerph16224420 (PMC6888634; doi:10.3390/ijerph16224420)
Supplement: Supplementary file 1 [file ijerph-16-04420-s001.pdf]

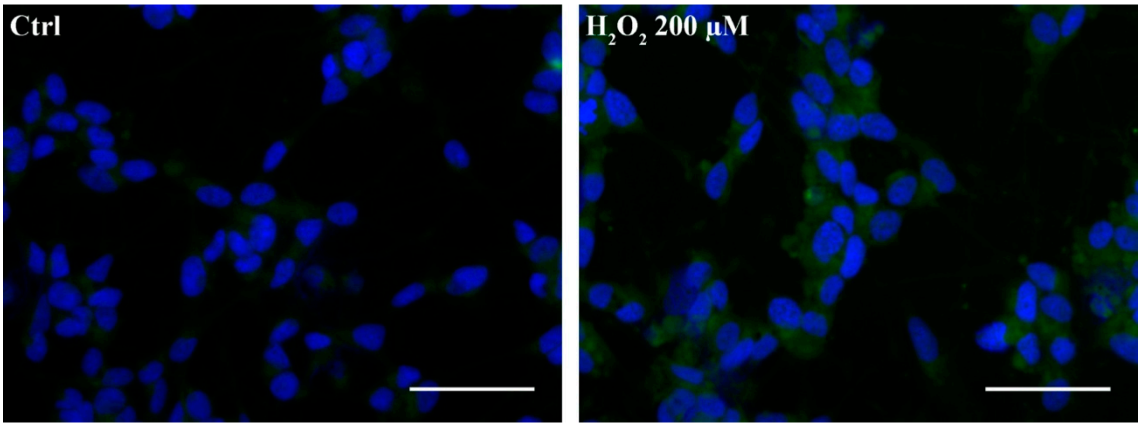

Figure S1. Immunofluorescence staining of ROS positive control. ROS expression was quantified by the 2′,7′- dichlorodihydrofluorescein diacetate (CM-H_2DCFDA) probe. The representative fluorescent images show the increase of ROS production induced by H_2O_2 200 μM exposure after 24 h. Total magnification: 400×. Scale bar: 50 μm.
